# Combinatorial Synthesis of Magnesium Tin Nitride Semiconductors

**DOI:** 10.1021/jacs.0c02092

**Published:** 2020-04-11

**Authors:** Ann L. Greenaway, Amanda L. Loutris, Karen N. Heinselman, Celeste L. Melamed, Rekha R. Schnepf, M. Brooks Tellekamp, Rachel Woods-Robinson, Rachel Sherbondy, Dylan Bardgett, Sage Bauers, Andriy Zakutayev, Steven T. Christensen, Stephan Lany, Adele C. Tamboli

**Affiliations:** † Materials and Chemistry Science and Technology Directorate, National Renewable Energy Laboratory, Golden, Colorado 80401, United States; ‡ Department of Physics, Colorado School of Mines, Golden, Colorado 80401, United States; § Applied Science and Technology Graduate Group, University of California at Berkeley, Berkeley, California 94720, United States; ∥ Energy Technologies Area, Lawrence Berkeley National Laboratory, Berkeley, California 94702, United States; ⊥ Department of Metallurgical and Materials Engineering, Colorado School of Mines, Golden, Colorado 80401, United States

## Abstract

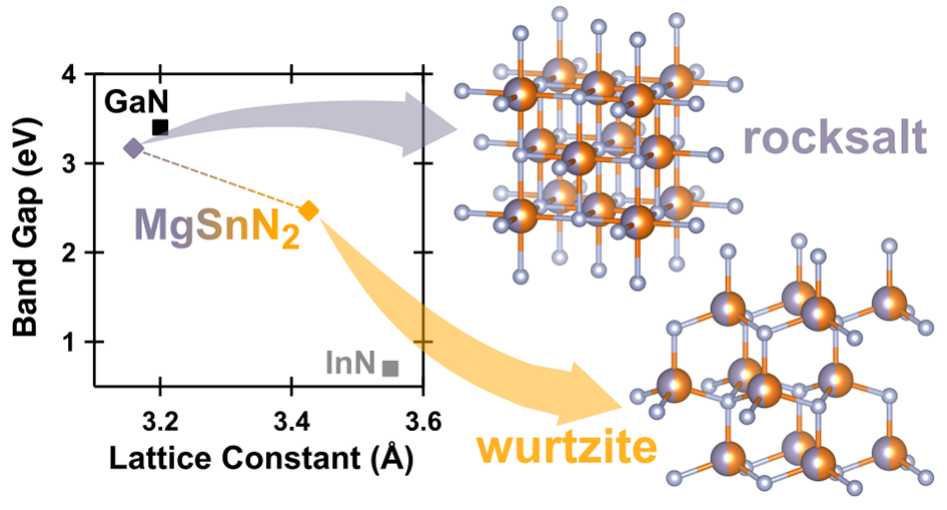

Nitride materials feature strong chemical bonding character that leads to unique crystal structures, but many ternary nitride chemical spaces remain experimentally unexplored. The search for previously undiscovered ternary nitrides is also an opportunity to explore unique materials properties, such as transitions between cation-ordered and -disordered structures, as well as to identify candidate materials for optoelectronic applications. Here, we present a comprehensive experimental study of MgSnN_2_, an emerging II–IV–N_2_ compound, for the first time mapping phase composition and crystal structure, and examining its optoelectronic properties computationally and experimentally. We demonstrate combinatorial cosputtering of cation-disordered, wurtzite-type MgSnN_2_ across a range of cation compositions and temperatures, as well as the unexpected formation of a secondary, rocksalt-type phase of MgSnN_2_ at Mg-rich compositions and low temperatures. A computational structure search shows that the rocksalt-type phase is substantially metastable (>70 meV/atom) compared to the wurtzite-type ground state. Spectroscopic ellipsometry reveals optical absorption onsets around 2 eV, consistent with band gap tuning via cation disorder. Finally, we demonstrate epitaxial growth of a mixed wurtzite-rocksalt MgSnN_2_ on GaN, highlighting an opportunity for polymorphic control via epitaxy. Collectively, these findings lay the groundwork for further exploration of MgSnN_2_ as a model ternary nitride, with controlled polymorphism, and for device applications, enabled by control of optoelectronic properties via cation ordering.

## Introduction

Metal nitrides are ubiquitous in industrial applications^1−4^ and the technological importance of specific nitrides, such as III–N semiconductors,^5^ has encouraged a search for related compounds. II–IV–N_2_ compounds are III–N analogs where each pair of III^3+^ atoms (i.e., Ga) is substituted by one II^2+^ (Mg or Zn) and one IV^4+^ (Si, Ge, or Sn). These ternaries have band gaps and lattice parameters comparable with III-Ns, but are uniquely tunable: they form with cation ordering or with various degrees of disorder that modifies optoelectronic properties without a concomitant change in stoichiometry.^6^ Despite the scientific and technological potential represented by this tunability, II–IV–N_2_ compounds have only recently become a focus in semiconductor synthesis.^6−9^

Understanding the influence of cation ordering on II–IV–N_2_ materials characteristics would enable new property control across ternary and multinary semiconductors. With complete cation disorder, II–IV–N_2_ compounds effectively share the wurtzite structure of III-Ns; cation ordering modifies that structure, forming an orthorhombic supercell without changing the effective lattice parameter.^6^ The transition from order to disorder has been predicted to reduce the band gap (*E*_g_) of II–IV–N_2_ compounds by up to 1 eV.^10−12^ Although an experimental reduction has been observed for the related ZnSnP_2_,^13^ cation ordering in II–IV–N_2_ compounds has proven difficult to control and distinguish from other phenomena (e.g., oxygen incorporation^14^ or degenerate doping).^15^ These investigations are further complicated by low-temperature thin-film syntheses that kinetically trap material in a cation-disordered state, and by material-specific difficulties measuring cation ordering in the most-studied II–IV–N_2_ compounds, ZnGeN_2_^14,16^ and ZnSnN_2_.^10^

MgSnN_2_ is an excellent candidate material for observation of cation ordering due to the substantially different X-ray scattering factors of the cations,^17,18^ but there are few calculations of its properties. Initial theoretical work, assuming that MgSnN_2_ would share the wurtzite-type lattice of Zn-based II–IV–N_2_ compounds, predicted *E*_g_ ≈ 3.5 eV, too large for many optoelectronic applications.^19,20^ Recently, the use of improved basis sets has lowered this prediction to ∼2.3 eV,^21^ increasing interest in MgSnN_2_. There are a limited number of known semiconductors with *E*_g_ in the 1.8–2.5 eV range that can be epitaxially integrated with other materials, hindering the development of tandem photovoltaics and green light-emitting diodes. Other recent computational work has predicted MgSnN_2_ to be thermodynamically stable at room temperature and pressure,^22^ as well as stable against decomposition into the parent binary compounds,^21^ making it an attractive target for both fundamental exploration and device applications.

There are two recent reports on the synthesis of MgSnN_2_: thin-film deposition by molecular beam epitaxy^23^ and high-pressure bulk metathesis.^24^ A stoichiometric wurtzite-type phase is reported in the thin-film study, although no X-ray diffraction or elemental characterization is shown.^23^ Surprisingly, the high-pressure synthesis resulted in rocksalt MgSnN_2_,^24^ which had not been previously predicted and warrants in-depth investigation. While these studies provide an initial view of MgSnN_2_, they are focused on single cation stoichiometries and much remains to be learned about the optoelectronic properties of MgSnN_2_. A more expansive approach is required to understand MgSnN_2_ across its cation phase space and to contextualize its properties with other II–IV–N_2_ compounds.

Here, we present the synthesis of thin-film MgSnN_2_ across a range of cation compositions and temperature by combinatorial radio frequency (RF) cosputtering, providing a detailed exploration of this material’s cation phase space with corroboration from first-principles calculations. We observe wurtzite MgSnN_2_ up to 500 °C, as well as formation of the secondary rocksalt phase at Mg-rich compositions and temperatures at or below 100 °C. We performed a structure search and confirmed that an ordered wurtzite structure (*Pna*2_1_) is the ground state, while the lowest energy rocksalt structure (*P*2*/c*) is predicted to be metastable with an energy of 70 meV/at above *Pna*2_1_. While these phases have predicted *E*_g_ of 2.47 and 3.17 eV, respectively, spectroscopic ellipsometry modeling shows that the synthesized MgSnN_2_ has absorption onsets ≤2.0 eV, consistent with band gap reduction due to cation disorder. We also demonstrate epitaxial growth of both phases of MgSnN_2_ on GaN, a promising achievement for potential device applications which further highlights the need to understand phase formation dynamics in this material. This work provides an in-depth exploration of MgSnN_2_, a powerful model system for understanding and potentially controlling cation ordering and *E*_g_ in a broad array of multinary compounds. The observed wurtzite-rocksalt phase crossover may be relevant to other multinary compounds, opening the door to optoelectronic property control through both cation ordering and phase formation in a single material.

## Materials and Methods

### Combinatorial Library Deposition

Combinatorial RF cosputtering is uniquely situated to enable the deposition of new nitrides and has been used in recent work on II–IV–N_2_ compounds^25−27^ and metastable nitrides^28−30^ including Mg-containing compounds.^31,32^ In this study, 14 combinatorial RF thin-film depositions were performed using an AJA International ATC 2200-V sputtering system equipped with infrared heaters. Prior to deposition, the chamber was evacuated to a base pressure between 2–8 × 10^–8^ Torr. The working deposition pressure was 12.5 ± 0.3 mTorr, provided by 15 sccm N_2_ and 5 sccm Ar. The N_2_ flow passed through an electron-cyclotron resonance (ECR) plasma source set at 150 W, providing activated nitrogen. Sputtered material came from 3″ Mg and Sn targets (Kurt J. Lesker Company, 99.98% and 99.998% purities) angled at 45° to the stationary substrate normal, providing a gradient in cation fluxes and therefore MgSnN_2_ composition.

2″ × 2″ MgSnN_2_ sample libraries were deposited on *p*-type Si wafers with native oxide (University Wafer), 100 nm thermal oxide on Si (University Wafer), 1 cm × 1 cm glassy carbon (HGW GmbH, Germany) or on GaN (grown on Al_2_O_3_).^33^ All samples were rinsed in acetone and isopropanol and dried with N_2_ immediately before loading into the deposition system. Prior to each deposition, the targets were sputtered for at least 30 min with the shutters closed. Depositions ran between 45 and 120 min at temperatures between ambient (no temperature set point) and 575 °C (temperatures previously calibrated with infrared heating without sputtering). Sputter gun powers were adjusted to vary the range of cation compositions in the final library and ranged from 134–180 W for Mg and 30–66 W for Sn.

### Library Characterization

Following deposition, the 2″ × 2″ sample areas were mapped as 4 × 11 point sample “libraries”. Each library was characterized using a suite of mapping techniques and point characterization methods; mapping data was processed using CombIgor, a custom *Igor Pro* (WaveMetrics, Lake Oswego, OR, U.S.A.) package.^34^ Wide-angle X-ray scattering was performed on beamline 1–5 with an incident energy of 12.7 keV at the Stanford Synchrotron Radiation Lightsource (SSRL) for select samples. X-ray diffraction (XRD) mapping was performed using a Bruker D8 Discover equipped with an area detector, using θ-2θ geometry and Cu Kα radiation, and General Area Detector Diffraction System software. Film thicknesses were measured using a Dektak profilometer and were between 150 and 500 nm depending on growth time.

Cation composition maps were collected using a Bruker M4 Tornado Micro-XRF spectrometer, using a Rh excitation beam and two detectors. Rigorous characterization of cation composition (atom fraction, here Mg/(Mg+Sn)) is often neglected in studies of multinary materials and is particularly important for combinatorial syntheses. It is particularly important to characterize Mg content when comparing samples deposited at different temperatures as Mg has a high vapor pressure.^35^ However, Mg content is particularly difficult to measure due to its small atomic number. XRF is a sensitive method for characterizing elemental compositions of thin-films but only when interactions between the fluoresced photons and the matrix are minimized (when films are thin) and calibrated using another technique.^36^ Further details are given in the Supporting Information and Figure S1.

Spectroscopic ellipsometry was performed on a single row of each sample (11 points) using a J.A. Woollam Co. M-2000 variable angle ellipsometer at angles close to the Brewster angle of Si: 65°, 70°, and 75°. The CompleteEASE software (version 5.08) was used to do the modeling.^37^ The samples were modeled by fitting the imaginary part of the dielectric function with Tauc-Lorentz oscillators. For samples that exhibited free carrier absorption at low energies a Drude oscillator was also incorporated in the model.

### Single-Point Characterization

Single-point Rutherford backscattering spectroscopy (RBS) was used to calibrate XRF cation compositions and measure anion compositions in MgSnN_2_. RBS was carried out across the combinatorial spread using a model 3S-MR10 RBS system from National Electrostatics Corporation. The RBS beam consisted of 2 MeV alpha particles, and the total accumulated charge was 80 or 160 μC. The RBS detector was mounted at a 168° backscatter configuration, and a secondary, moveable detector was set at 140° when used. RBS spectra analysis was performed using the RUMP data analysis software.^38^

Scanning electron microscopy (SEM) images were collected using a JEOL JSM-7000F field emission SEM using 10 kV accelerating voltage and 10 mm working distance. Electronic transport measurements were performed using a Lakeshore 8425 Hall probe equipped with a 2T superconducting magnet and variable temperature control. Low-temperature (40 K) measurements were made on ca. 5 × 5 mm chips of combinatorial libraries in the van der Pauw configuration, with some composition variation across the samples. Pole figures were collected using a Rigaku Smartlab diffractometer using a Ge (220) × 2 monochromator.

### Computational Methods

First-principles density functional (DFT) and many-body perturbation theory calculations were performed with the VASP code.^39−41^ Crystal structure prediction was performed using the kinetically limited minimization (KLM) approach.^29^ For efficient structure sampling, we employed the standard generalized gradient approximation (GGA),^42^ and to obtain more accurate lattice parameters, we used the strongly constrained and appropriately normed (SCAN) meta-GGA.^43^ Total energy calculations in the random phase approximation (RPA)^41^ were performed upon the SCAN relaxed structures and are reported in Table 1. (A comparison of RPA and DFT energies is given in Table S1.) The energy cutoffs, k-point density, and number of bands for the RPA were increased as needed to ensure convergence better than 1 meV/at for the absolute total energies. Electronic structure and band gap calculations were performed using the GW approximation following previous methods.^44^ The optical absorption coefficient was calculated from the frequency dependent dielectric matrix in the independent particle approximation.^45^

## Results and Discussion

### Composition and Phase Space

**Figure 1:**
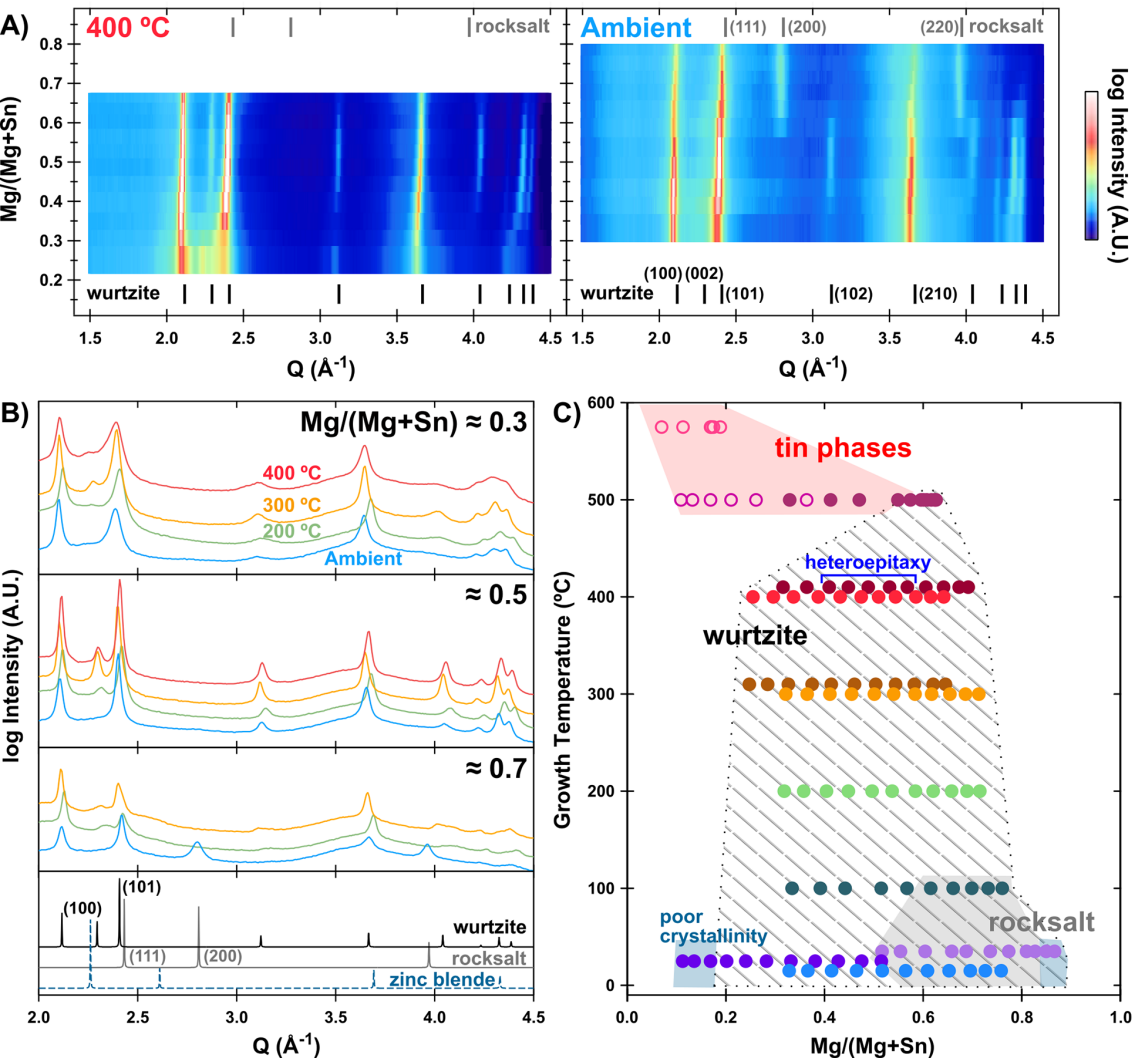
Structural analysis and composition map of MgSnN_2_. (A) Synchrotron XRD heatmaps of MgSnN_2_ sample libraries grown at 400 °C and ambient deposition temperature. Reference diffraction peak positions are given for disordered wurtzite (black) and disordered rocksalt (gray) phases. (B) Synchrotron XRD for four MgSnN_2_ sample libraries at different Mg/(Mg + Sn). Reference patterns are given for the disordered wurtzite and rocksalt phases, with the reference pattern for the absent zinc blende phase (see discussion below) given with a dashed line. In panels A and B, slight displacements of the experimental peaks relative to the predicted positions are a result of sample misalignment. (C) Experimental phase map of MgSnN_2_ with all compositions measured by XRF calibrated with RBS. Each sample library has a unique color. Open circles at high temperature indicate libraries with low Mg absorption (to the extent that continuous films did not form). The area where the wurtzite phase appears is indicated with shaded lines. While the rocksalt phase (gray shading) is present at low temperatures and Mg-rich conditions, there are no crystalline samples in that region where the wurtzite (100) peak is absent, indicating a two-phase region. The tin phases (red shading) at high temperature are either metallic Sn or Sn_3_N_4._ The two shaded blue areas represent poorly crystalline regions described in the text. The blue bracket denotes samples with heteroepitaxial alignment to GaN, discussed later.

Exploration of MgSnN_2_ by combinatorial cosputtering began with deposition of sample libraries on Si substrates at temperatures ranging from no intentional heating (hereafter referred to as ambient) to 400 °C. Synchrotron XRD heatmaps of two libraries are shown in Figure 1A. At 400 °C, peaks corresponding to the predicted MgSnN_2_ cation-disordered wurtzite structure at all magnitudes of the scattering vector *Q* are apparent across the library. No superlattice peaks (*Q* < 2.0) or peak splitting are observed, indicating that this material is cation disordered.^6^ The ambient temperature library also has a wurtzite phase present, 0.3 < Mg/(Mg + Sn) < 0.6, although no (002) peak is observed as a result of strong texturing in the (100) and (101) directions. However, at Mg/(Mg + Sn) > 0.54, another phase appears as many wurtzite peaks disappear, though the (100) and (210) reflections remain. The wurtzite (101) peak shifts to higher *Q* at this point and is now indexed to the rocksalt (111) reflection.

To examine the effect of cation stoichiometry on phase formation, synchrotron XRD for single stoichiometries across libraries grown at different temperatures are compared in Figure 1B. At Mg/(Mg + Sn) ≈ 0.5, all samples are highly crystalline but have different texturing, as seen by changes in the prominence, but not peak shape, of the wurtzite (002) and higher Q reflections across the four samples. Peaks for all samples are less well-resolved at both Mg/(Mg + Sn) ≈ 0.3 and ≈ 0.7, indicating a reduction in crystallinity or smaller grain sizes off of stoichiometry. At Mg/(Mg + Sn) ≈ 0.3, the 300 °C sample retains the most crystallinity with good peak resolution at high Q. At Mg/(Mg + Sn) ≈ 0.7, rocksalt peaks are evident in the ambient temperature sample, while the 200 and 300 °C samples show only the wurtzite phase with texturing in the (100) and (101) directions. The same trends were observed directly using SEM (Figure S2).

Additional libraries were deposited on Si substrates to define the limits of the phase space (each library is represented by a different color in Figure 1C). Three libraries were deposited at 500 °C and above; although (002)-textured, wurtzite MgSnN_2_ was grown at 500 °C with increased Mg power, only Sn-based phases were observed above that temperature or with the original sputter target powers. Two more libraries were deposited at ambient temperature to explore the entire range of cation compositions. Reduced Sn flux gave a higher Mg/(Mg + Sn) overall and a library where the rocksalt phase was present at all compositions; the samples with the highest Mg content largely oxidized before XRD could be performed, although some wurtzite peaks were still visible in this poorly crystalline region. Increasing Sn flux at ambient temperature resulted in a mostly wurtzite library but with poor crystallinity at the lowest Mg/(Mg + Sn). While immediate oxidation and delamination were observed for the high Mg-content ambient temperature sample library (light purple circles in Figure 1C), overall the synthesized MgSnN_2_ libraries were very stable, with little or no change by XRD over time. After an extended period, the highest Mg-content points on several libraries did visually change, suggesting some oxidation (Figure S3).

Although crystallinity and texturing vary across the sample set, it is clear that wurtzite MgSnN_2_ forms with a large tolerance to off-stoichiometry, being present from 0.25 < Mg/(Mg + Sn) < 0.75 up to 400 °C and at 500 °C in a narrower range of stoichiometries. This stoichiometry window is consistent with other II–IV–N_2_ compounds deposited by combinatorial sputtering but with a higher maximum temperature due to the lower volatility of Mg^35^ than Zn.^26^ The rocksalt phase of MgSnN_2_ forms consistently with Mg/(Mg + Sn) ≥ 0.55 at low temperature but is never phase-pure, as the (100) peak of the wurtzite phase is always present. The previously reported high-pressure rocksalt phase forms at a similar composition, Mg/(Mg + Sn) = 0.54,^24^ to that observed in this study, despite the low-pressure growth environment used here. The existence of multiple MgSnN_2_ phases is in contrast to well-studied II–IV–N_2_ compounds that only form in wurtzite-type phases^46^ and to recently synthesized Mg-based transition metal rocksalt compounds.^31^

### Computational Investigation

**Figure 2:**
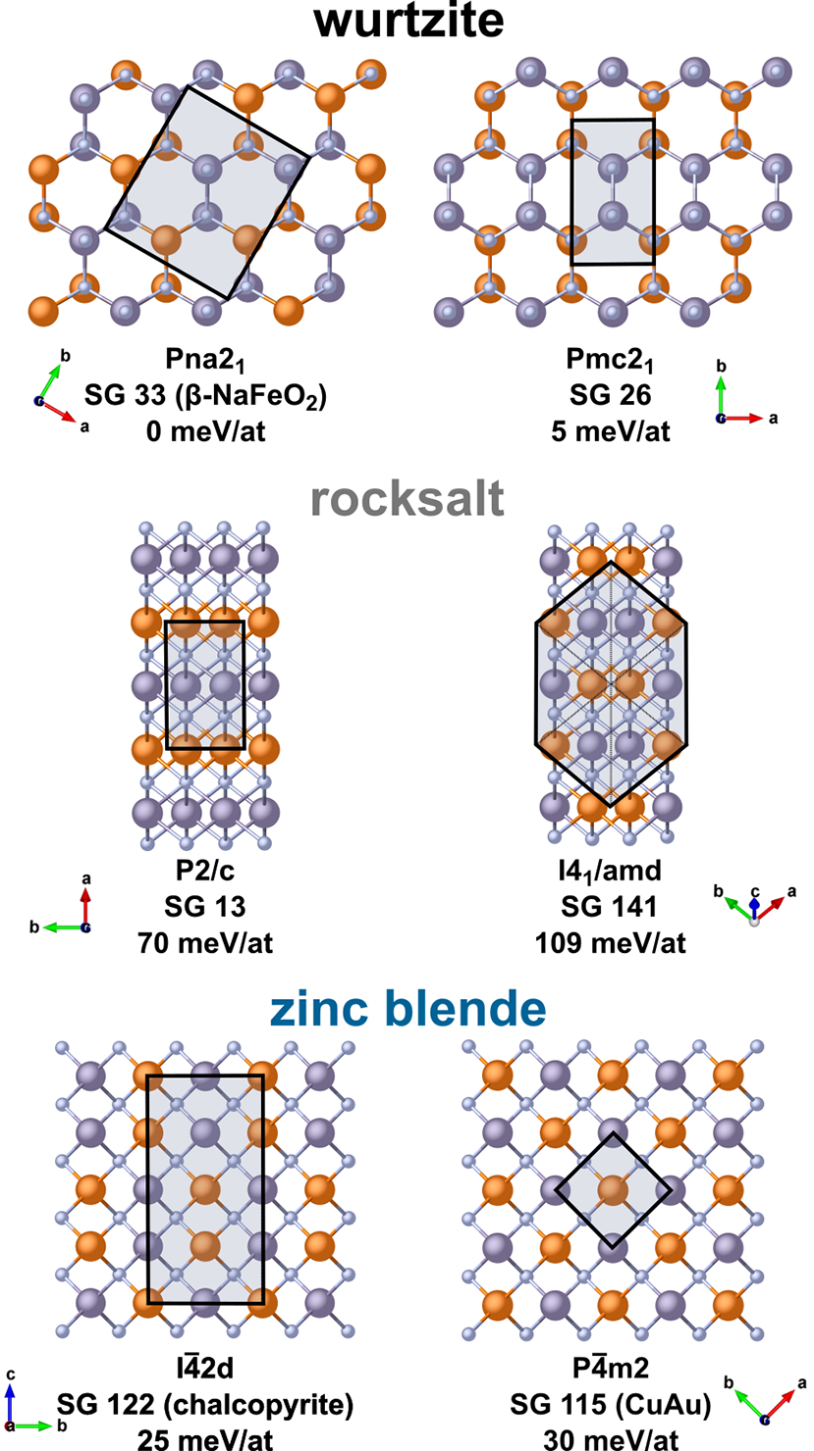
Predicted cation-ordered structures for MgSnN_2_, grouped by the binary structure from which they are derived, with SG name and number, common name if available, and energy above the ground state (taken to be Pna2_1_). Mg atoms are orange, while Sn are gray. The structures have been oriented to the same presentation for each lattice type to facilitate comparisons. Unit cells are indicated with shading. For the prototypical structures discussed here, Crystallographic Information Files (CIF) were generated from the SCAN relaxed structures using the FINDSYM software.^47^ The CIF files are available in the Supporting Information.

Given the coformation of wurtzite and rocksalt MgSnN_2_, the formation energetics of both phases must be understood. In order to identify the most energetically favorable structures, we performed structure prediction based on DFT energies, using the KLM approach that has recently been applied to other stable and metastable ternary nitrides.^29^ This structure sampling found six well-known structure types representing ordered supercells of wurtzite, zinc blende, and rocksalt structures; these structures are shown in Figure 2 (with predicted diffraction patterns in Figure S4). The predicted structures are all cation-ordered, as disorder generally increases formation enthalpy.

In previous computational work, the ground state of MgSnN_2_ has been assumed to be identical to other II–IV–N_2_ compounds, including ZnGeN_2_, i.e., a cation-ordered wurtzite lattice with an orthorhombic primitive cell: *Pna*2_1_, space group (SG) no. 33.^19^ We confirm this assumption, as the *Pna*2_1_ structure emerged as the lowest energy structure, with the next lowest being *Pmc*2_1_ (SG 26), another orthorhombic, ordered wurtzite structure containing exclusively octet-rule-preserving coordination motifs (each N bound to only two Mg and two Sn atoms). The energy difference between the wurtzite-type structures is 5 meV/atom (see Figure 2), larger than that in ZnSnN_2_ (1 meV/at) but less than that in ZnGeN_2_ (27 meV/at). In addition to the wurtzite-type structures, DFT identified supercells of zinc blende and rocksalt-based structures. The zinc blende lattice supports two octet-rule-conserving motif structures, *I*4̅2*d* (SG 122) and *P*4̅*m*2 (SG 115), which are the known chalcopyrite and CuAu-type cation-ordered zinc-blende structures, respectively. In the octahedrally coordinated rocksalt lattice, charge-balanced N–Mg_3_Sn_3_ motifs form *P*2/*c* (SG 13) and *I*4_1_*/amd* (SG 141) structures, recently identified as the ground states of MgTiN_2_ (a slight symmetry reduction from *R*3̅*m*, SG 166, the α-NaFeO_2_ structure) and MgZrN_2_, respectively.^31^ The rocksalt structures differ in the clustered vs dispersed arrangement of Mg and group IV atoms within the N-centered octahedron.

The calculated total energies show that the wurtzite-type structures of MgSnN_2_ are the most energetically favorable, followed by the zinc blende-type, whereas the rocksalt-type structures are considerably higher in energy (Figure 2). As face-centered cubic structures, zinc blende and rocksalt produce similar diffraction patterns. However, the large difference in lattice constants makes them easily distinguishable, and the diffraction data shown in Figure 1 is indicative of the formation of the disordered rocksalt, not the zinc blende, as a metastable phase. The 20% smaller unit cell volume of the rocksalt structure compared to the wurtzite and zinc blende structures suggests that MgSnN_2_ could be stabilized as a high-pressure phase (as recently observed)^24^ or possibly in nonequilibrium thin-film growth, e.g., under considerable local stress. Large amounts of strain are known to be introduced in high growth-rate processes such as the combinatorial cosputtering used in this study.^48^ A rocksalt phase of MgSiN_2_ has been observed following high-pressure treatment of wurtzite MgSiN_2_,^49^ suggesting that this phase may be accessible across the Mg-containing II–IV–N_2_ materials. This hypothesis is bolstered by the fact that Mg more readily forms compounds with octahedral coordination than Zn.^50^

Although cation-ordered wurtzite MgSnN_2_ has not been experimentally documented, the DFT-predicted wurtzite-type structures can give insight into the formation of the cation-ordered phases. Calculated distortions of the *Pna*2_1_ orthorhombic phase, measured by comparing lattice parameters *a* and *b*, have been proposed as a proxy for the tendency toward cation ordering in II–IV–N_2_ compounds.^6^ We find for MgSnN_2_*a/b* = 0.993 × √3, i.e., 0.7% smaller than in undistorted wurtzite. For comparison, ZnGeN_2_, which has been observed in the ordered *Pna*2_1_ structure, the distortion is −2.1%. In ZnSnN_2_, where ordering has not conclusively been confirmed, the distortion is only +0.2%.^11^ For MgSnN_2_, the distortion of −0.7% suggests that cation ordering may be achievable and observable in this material if the right growth conditions can be found. Experimental observation of (long- or short-range) ordering via diffraction could be facilitated in MgSnN_2_ by the large differences between the Mg and Sn scattering factors, whereas the similarity between Zn and Ge has hampered the analysis of cation ordering in related materials.^16^

Another comparison of lattice parameters *a′* = √(*ab*/2√3) (i.e., the average wurtzite basal plane lattice constant *a*′ calculated from the orthorhombic *a* and *b* constants) and *c′ = c* in the *Pna*2_1_ structure reveals a difference between Zn- and Mg-based II–IV–N_2_ compounds. The ideal binary wurtzite has *c/a*′ = 1.633; this is also the value for ZnGeN_2_, while ZnSnN_2_ is slightly smaller, *c/a*′ = 1.622. MgSnN_2_, on the other hand, has *c/a*′ = 1.598, 2.2% smaller than the ideal and MgSiN_2_, which is also known to have accessible wurtzite and rocksalt phases, has *c/a*′ = 1.592, 2.5% smaller. It is possible that the deviation of the *c*/*a*′ ratio from ideal wurtzite could be correlated with the propensity to form a rocksalt phase.

### Oxygen Incorporation

We have proposed that high pressure, either directly or from high local strain, stabilizes rocksalt MgSnN_2_. While the cosputtering environment may be sufficiently away from equilibrium to provide this stabilization, another factor may contribute: the presence of oxygen. We previously developed a defect model for ZnSnN_2_ suggesting the formation of (ZnSnN_2_)_1–*x*_(ZnO)_2*x*_ alloys within their shared wurtzite structure.^51^ An analogous mechanism could facilitate a wurtzite-rocksalt crossover here, as MgO has a strong energy preference for the rocksalt structure.^52^ We grew thin MgSnN_2_ libraries at ambient and 300 °C to investigate the presence of oxygen via RBS; glassy carbon was used as a substrate to reduce convolution of the film and substrate signals. Both libraries show substantial oxygen incorporation throughout the films: at 300 °C, average anion composition O/(N + O) was 0.15 ± 0.02, and at ambient, O/(N + O) = 0.31 ± 0.03 (see the SI). In both cases, O/(N + O) was slightly reduced in Mg-poor regions. Although ex situ oxidation is likely, RBS indicates oxygen throughout the thickness of the film, suggesting oxygen incorporation during growth, and we have not conclusively identified its source.

The presence of oxygen does not conclusively indicate the formation of a mixed nitride-oxide phase, but the high concentration of oxygen, especially in regions where rocksalt is observed, does support the hypothesis that rocksalt-derived MgSnN_2_ is templated by MgO, although no MgO peaks are observed by diffraction (MgO can be sputtered at low temperatures without crystallizing).^53^ Kawamura et al. also noted oxygen incorporation in their high-pressure rocksalt, suggesting that it performed a charge neutralization or octet-rule conservation function^24^ similar to that in the (ZnSnN_2_)_1–*x*_(ZnO)_2*x*_ system. While we have been interested in investigating cation order in wurtzite MgSnN_2_ by eliminating the rocksalt phase, it is possible that rocksalt MgSnN_2_ could be isolated via growth directly on MgO, enabling investigation of its unique optoelectronic properties.

### Optoelectronic Properties

The calculated optoelectronic properties for the six cation-ordered MgSnN_2_ structures are presented in Table 1. The direct band gaps of *E*_g_ = 2.47 and 2.34 eV for the ordered wurtzite structures agree broadly with recent calculations by Lyu and Lambrecht (*E*_g_ = 2.28 eV for *Pna*2_1_).^21^ The zinc blende structures have similar *E*_g_ which are also direct. The rocksalt structures have larger gaps of *E*_g_ = 3.17 and 2.93 eV that have an indirect or forbidden character, as seen by the difference between the fundamental gap and the absorption threshold for α = 10^3^ cm^–1^ (see Table 1). The electron effective masses (band effective mass) are m*_e_/*m*_0_ = 0.2 (where *m*_0_ is the free-electron rest mass) for all structures, indicating good electron transport. To understand the trend in the hole effective masses, we evaluated the density of states effective mass, which integrates over band and Brillouin zone degeneracies and is always larger than the individual band masses. The value m*_h_/*m*_0_ = 2.4 found for *Pna*2_1_, the ground state, is comparable to that of GaN, but the rocksalt structures exhibit considerably heavier hole masses. The dielectric constants for the rocksalt structures are also much larger than for the tetrahedral structures, a typical feature for rocksalt nitrides.^31^ Calculated dielectric functions for the six cation-ordered MgSnN_2_ structures are presented in Figure S5. It can be expected that the band gaps of these materials further increase with O incorporation, which could make rocksalt (MgSnN_2_)_1–*x*_(MgO)_2*x*_ alloys an interesting subject for future studies of wide gap materials. While defect calculations are beyond the scope of this work, the formation of a Mg–O compensating defect is likely, similar to the behavior of Zn–O defects in ZnSnN_2_.^51^

**Table 1:** Predicted Structures, Polymorph Energies, Band Gaps, Absorption Thresholds (α = 10^3^ cm^–1^) with Indirect/Forbidden Transitions Marked with *, Hole Effective Masses, and Dielectric Constants, Including Both Electronic and Ionic Contributions

			optoelectronic structure			
polymorph type	space group no.	relative energies (RPA) (meV/at)	*E*_g_ (eV)	*E* at 10^3^	m*_h/_*m*_0_	ε
wurtzite	33	0	2.47	2.60	2.4	9.1
	26	5	2.34	2.45	3.0	9.1
zinc blende	122	25	2.33	2.42	3.7	9.1
	115	30	2.13	2.22	4.2	9.3
rocksalt	13	70	3.17	3.56*	5.5	22.7
	141	109	2.93	3.24*	7.7	27.5

**Figure 3:**
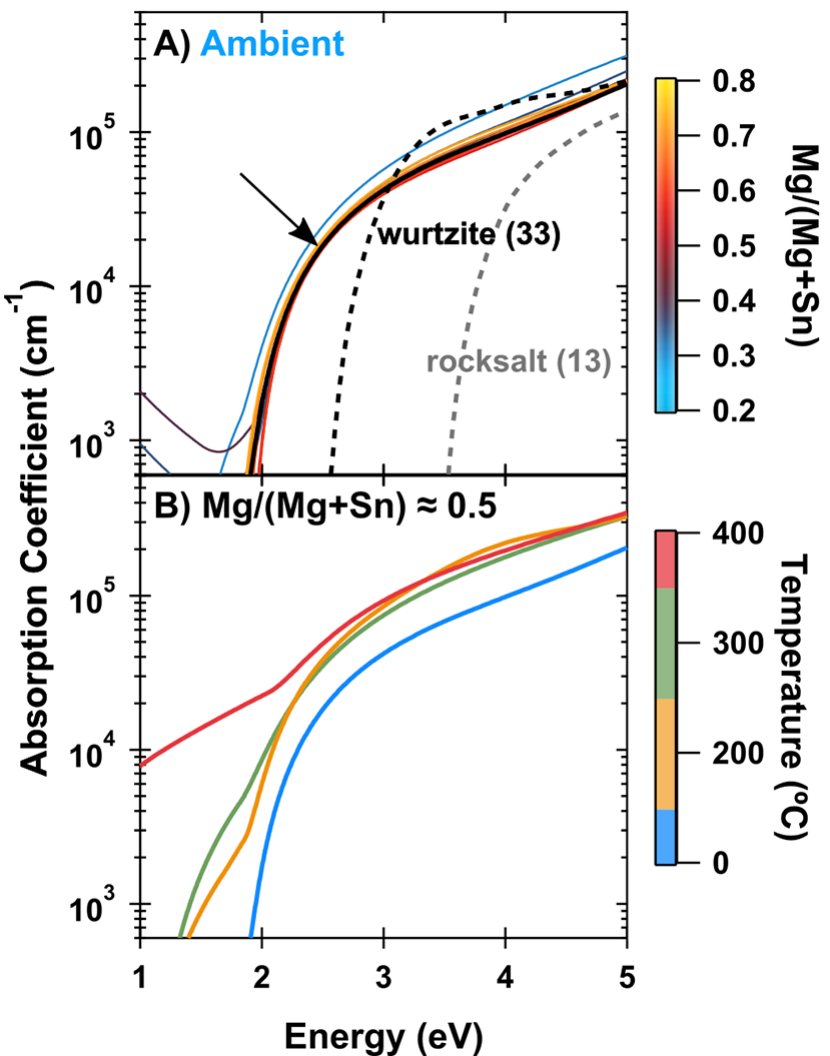
Absorption properties of MgSnN_2_ calculated from spectroscopic ellipsometry. (A) Ambient temperature MgSnN_2_ library with calculated absorption profiles for the lowest-energy cation-ordered wurtzite (SG 33) and rocksalt (SG 13) phases shown (dashed lines) for reference. The black arrow highlights sample with Mg/(Mg + Sn) ≈ 0.5. (B) Mg/(Mg + Sn) ≈ 0.5 sample absorption for a range of temperatures, including ambient. Colors are consistent with libraries in Figure 1.

We used spectroscopic ellipsometry to measure optical properties of four MgSnN_2_ libraries and fit the resulting data to obtain absorption coefficients. The modeled absorption for the whole composition range of the ambient temperature library (which has XRD shown in Figure 1A) is shown in Figure 3A. The absorption changes very little between Mg/(Mg + Sn) = 0.4 and 0.75, with a slight shift to lower energies at <0.4. Compared to the calculated absorption characteristics of the predicted ground state structure, the synthesized MgSnN_2_ films exhibit a reduced steepness of the absorption curve and an onset at about 0.5 eV lower energy. This observation is consistent with the effect expected for cation disorder, which has been investigated in more detail for ZnSnN_2_.^10^ While rocksalt MgSnN_2_ is observed in this sample (as shown in Figure 1A), there is limited change in the absorption coefficient across the Mg-rich regions of the library. This is a result of the coexistence of the rocksalt and wurtzite phases across that span, where the smaller, direct *E*_g_ of the wurtzite phase dominates the absorption over the larger, indirect *E*_g_ of the rocksalt. The low Mg/(Mg + Sn) samples for this ambient temperature library display free-carrier absorption tails, consistent with metallic defects and doping off-stoichiometry, although doping was not measured directly.

Modeled absorption characteristics of Mg/(Mg + Sn) ≈ 0.5 at four different temperatures are shown in Figure 3B. We attribute the increase in low-energy absorption with increasing temperature to a high defect density, which is corroborated by 10^19^–10^20^ cm^–3^*n*-type carrier concentrations measured via Hall effect at Mg/(Mg + Sn) ≈ 0.5 for libraries grown at 300 and 400 °C (Table S2). Although increasing growth temperature generally results in improved electronic properties, we observe both high oxygen content via RBS and the formation of secondary Sn phases (Figure 1C) at high temperature. Both a high oxygen concentration and metal impurities (which may be too dilute to resolve via XRD) would negatively impact electronic properties and are targets for further exploration in future work. Modeled dielectric functions and *n* and *k* values for the samples presented in Figure 3 are shown in Figure S6.

### Epitaxial Growth

**Figure 4:**
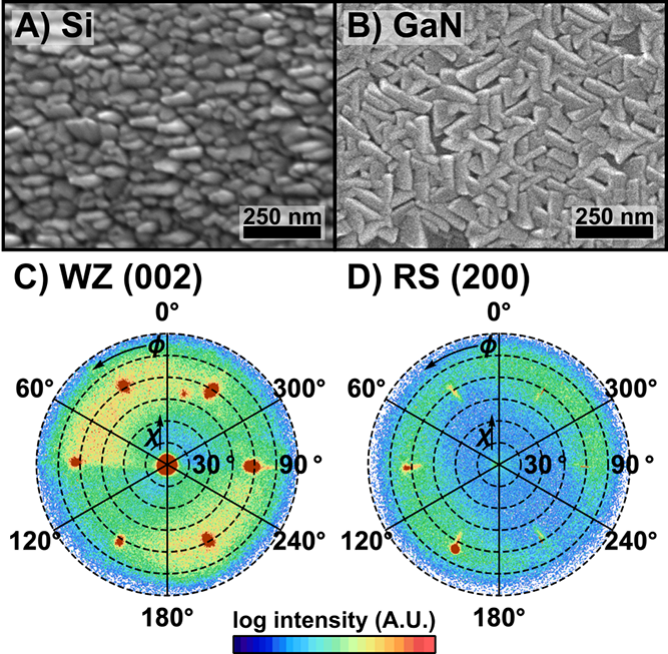
SEM images of MgSnN_2_ with Mg/(Mg + Sn) = 0.53 grown on (A) Si and (B) GaN substrates in the same deposition. Pole figures around (C) the MgSnN_2_ wurtzite (002) and (D) rocksalt (200) from the MgSnN_2_-on-GaN sample at Mg/(Mg + Sn) ≈ 0.5, showing the presence of both phases (variation in peak intensity is a result of slight sample misalignment).

The technological promise of MgSnN_2_, and of II–IV–N_2_ compounds as a whole, is partially because of its shared wurtzite structure with III-N compounds, which could enable heteroepitaxial devices. Cation-disordered wurtzite MgSnN_2_ has an *a-*lattice parameter of 3.426 Å, 7.3% mismatched from GaN. Despite this substantial mismatch, we deposited a combinatorial library of MgSnN_2_ directly on a GaN template^33^ at 400 °C and observed heteroepitaxial alignment to the substrate in the resulting material. SEM comparisons at Mg/(Mg + Sn) = 0.53 (Figure 4A,B) show similar grain sizes on GaN and on Si in the same deposition (two substrates were co-grown) but with clear alignment along crystallographic directions in the on-GaN sample which is lacking in the one on Si. The area XRD for the MgSnN_2_-on-GaN indicates heteroepitaxial alignment of material across 0.4 < Mg/(Mg + Sn) < 0.6 (noted in Figure 1C).

Two XRD pole figures were performed on the MgSnN_2_-on-GaN sample, at Mg/(Mg + Sn) ≈ 0.5, to conclusively identify alignment of the MgSnN_2_ to the substrate. The first pole figure, shown in Figure 4C, was performed on the wurtzite MgSnN_2_ (002) peak (*Q* = 2.2949). One peak is apparent at χ = 0°, consistent with alignment to the GaN (002); an additional set of peaks is apparent at χ = 61.4°, indicating another alignment of MgSnN_2_ to the substrate. The second pole figure, Figure 4D, was performed on the rocksalt MgSnN_2_ (200) peak (*Q* = 2.8082) and found six low-intensity peaks at χ ≈ 56°.

Together, the pole figures indicate that the MgSnN_2_ rocksalt phase is coincident with the wurtzite, albeit in small amounts, despite the high growth temperature where the rocksalt had not previously been observed. This is explained by the projection of the rocksalt (111) lattice plane on to the GaN hexagonal lattice. Unlike the 7.3% mismatch between the GaN and wurtzite MgSnN_2_ lattices, the distance between close-packed atoms in the rocksalt (111) planes is only ∼0.8% mismatched from GaN. This match promotes the formation of the rocksalt phase outside of the temperature and cation composition range which had previously been identified. The identification of rocksalt MgSnN_2_, even at high temperatures, highlights the fact that, in order to explore cation ordering in this material, the formation dynamics of its rocksalt phase must be investigated. Further work is crucial to obtain phase-pure material in order to understand cation ordering.

## Conclusion

We have presented the first comprehensive examination of the growth of thin-film MgSnN_2_, using the combinatorial approach to explore and characterize the phase space. Exploration of temperature and a wide range of cation compositions allowed us to identify a large parameter space for crystalline, cation-disordered wurtzite MgSnN_2_, as well as the co-occurrence of a cation-disordered rocksalt phase in Mg-rich samples at low temperatures. A DFT structure search shows that the rocksalt MgSnN_2_ is significantly metastable compared to the wurtzite-derived phases but may be stabilized by the strained environment created by cosputtering, as well as by oxygen on the anion site or other phenomena. Modeled spectroscopic ellipsometry shows that the MgSnN_2_ thin films have a lower optical absorption onset than the predicted *E*_g_ for either the cation-ordered wurtzite or rocksalt phase, consistent with other work on cation-disordered II–IV–N_2_ materials. MgSnN_2_ films grown at higher temperature display an increase in low-energy absorption, consistent with high electron densities measured via Hall effect. Finally, we have provided a conclusive demonstration of epitaxial MgSnN_2_, with a mixed wurtzite-rocksalt phase grown on GaN across a range of Mg/(Mg + Sn). Based on this work, MgSnN_2_ presents a unique opportunity to explore the factors controlling cation order in two separate crystal structures, where both the wurtzite- and rocksalt-derived phases show promise for heteroepitaxial integration.

## Supporting Information Available

Details of elemental characterization from Rutherford backscattering and X-ray fluorescence, scanning electron microscopy, detailed energy and optoelectronic calculations, calculated X-ray diffraction for disordered and ordered MgSnN_2_ structures, Hall effect measurements, calculated dielectric functions, modeled dielectric functions, and *n* and *k* values (PDF)
Calculated crystal structures for disordered wurtzite MgSnN_2_ (CIF)
Calculated crystal structures for SG 33 MgSnN_2_ (CIF)
Calculated crystal structures for SG 26 MgSnN_2_ (CIF)
Calculated crystal structures for disordered rocksalt MgSnN_2_ (CIF)
Calculated crystal structures for SG 13 MgSnN_2_ (CIF)
Calculated crystal structures for SG 141 MgSnN_2_ (CIF)
Calculated crystal structures for SG 166 MgSnN_2_ (CIF)
Calculated crystal structures for disordered zinc blende MgSnN_2_ (CIF)
Calculated crystal structures for SG 122 MgSnN_2_ (CIF)
Calculated crystal structures for SG 115 MgSnN_2_ (CIF)

## Supplementary Material

ja0c02092_si_001.pdf

ja0c02092_si_002.cif

ja0c02092_si_003.cif

ja0c02092_si_004.cif

ja0c02092_si_005.cif

ja0c02092_si_006.cif

ja0c02092_si_007.cif

ja0c02092_si_008.cif

ja0c02092_si_009.cif

ja0c02092_si_010.cif

ja0c02092_si_011.cif
