# Supplementary material for: Combinatorial Synthesis of Magnesium Tin Nitride Semiconductors
Source: J Am Chem Soc. 2020 Apr 11;142(18):8421–30. doi: 10.1021/jacs.0c02092 (PMC10905991; doi:10.1021/jacs.0c02092)
Supplement: Supplementary file 1 — ja0c02092_si_001.pdf [file ja0c02092_si_001.pdf]

Combinatorial synthesis of magnesium tin nitride semiconductors

Ann L. Greenaway,[1*] Amanda L. Loutris,[1] Karen N. Heinselman,[1] Celeste L. Melamed,[2,1] Rekha R. Schnepf,[2,1] M. Brooks Tellekamp,[1] Rachel Woods-Robinson,[1,3,4] Rachel Sherbondy,[5,1] Dylan Bardgett,[1] Sage Bauers,[1] Andriy Zakutayev,[1] Steven T. Christensen,[1] Stephan Lany,[1] Adele C. Tamboli[1,2]

[1] Materials and Chemistry Science and Technology Directorate, National Renewable Energy Laboratory, Golden, Colorado 80401, USA
[2] Department of Physics, Colorado School of Mines, Golden, Colorado 80401, USA
[3] Applied Science and Technology Graduate Group, University of California, Berkeley, CA 94720, USA
[4] Energy Technologies Area, Lawrence Berkeley National Laboratory, Berkeley, CA 94702, USA
[5] Department of Metallurgical and Materials Engineering, Colorado School of Mines, Golden, Colorado 80401, USA

*corresponding author, ann.greenaway@nrel.gov

Supporting Information

Composition and Phase Space

For cation composition, Rutherford backscattering spectroscopy (RBS), a low-throughput method, was used to characterize Mg/(Mg+Sn) for three points per sample library for four libraries to generate a calibration. Glassy carbon was used as a substrate to eliminate substrate overlap with the anion peaks (SI Figure 1A); additionally, a short growth time was used so that element signals (proportional to film thickness) would be well-resolved. X-ray fluorescence (XRF) mapping was used to quickly acquire composition spectra for full sample libraries. The XRF data was then converted to Mg/(Mg+Sn) using RBS results and integrated peak areas from XRF. Figure S1B gives a comparison of RBS and calibrated XRF data for two sample libraries. The XRF calibration somewhat underestimates Mg/(Mg+Sn) at the lowest Mg composition.

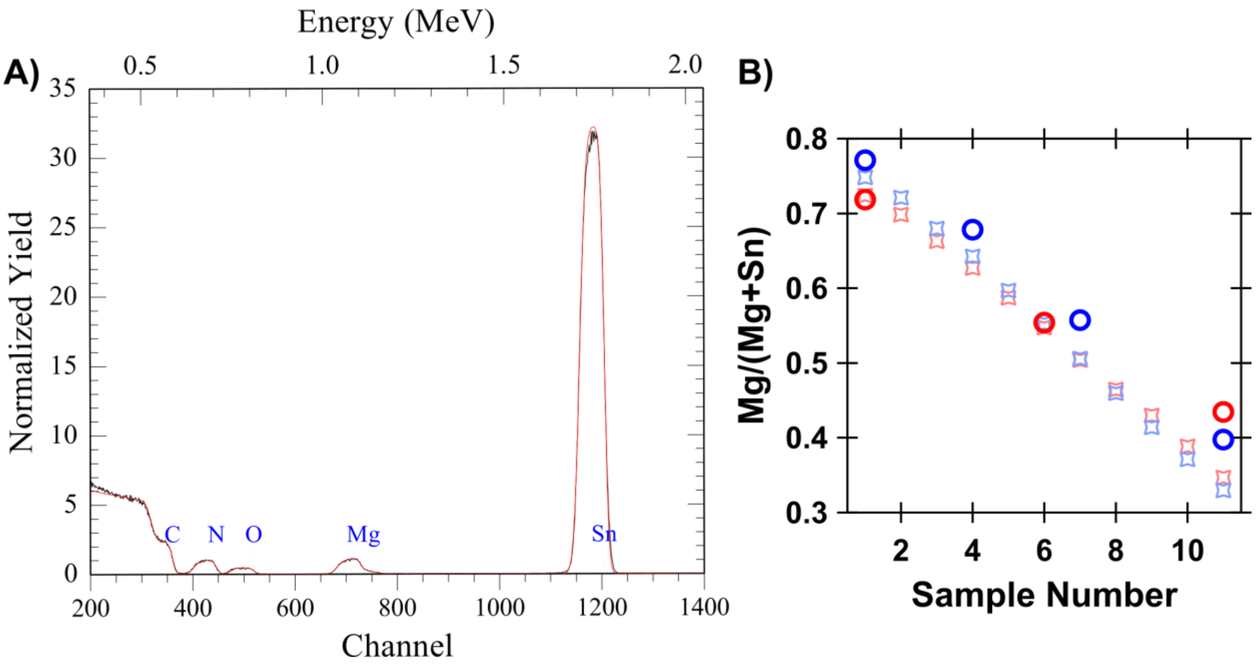

Figure S1: A) Example RBS data showing well-resolved signals for C, N, O, Mg, and Sn. Use of glassy carbon rather than Si eliminates overlap with the anion signals, simplifying analysis. B) Measured RBS (circles) and RBS-calibrated XRF (square shapes) data for two libraries, shown in separate colors.

The effect of changing stoichiometry on crystallinity can also be observed directly using SEM (Figure S2). At ambient temperature and below Mg/(Mg+Sn) = 0.5, the films have very small grains with no preferred orientation, while above Mg = 0.5, triangular crystallites are formed that increase in size with increasing Mg content. Such crystallites are consistent with the columnar grains often observed in thin films grown by sputtering methods. The Mg ≈ 0.5 composition can also be compared across a range of temperatures: above 300 ºC, grain size increases dramatically, with the largest grains observed at 400 ºC. The 500 ºC sample returns to columnar morphology, possibly correlated to the (002) texturing observed in that library.

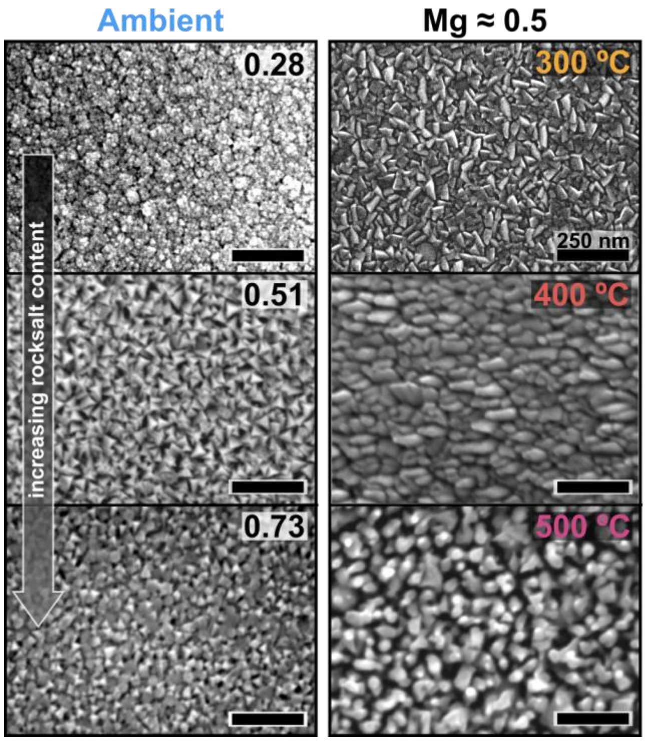

Figure S2: SEM comparisons of $MgSnN_2$ samples with different Mg/(Mg+Sn) deposited at ambient temperature (left column) and with Mg/(Mg+Sn) ≈ 0.5 deposited at different temperatures (right column).

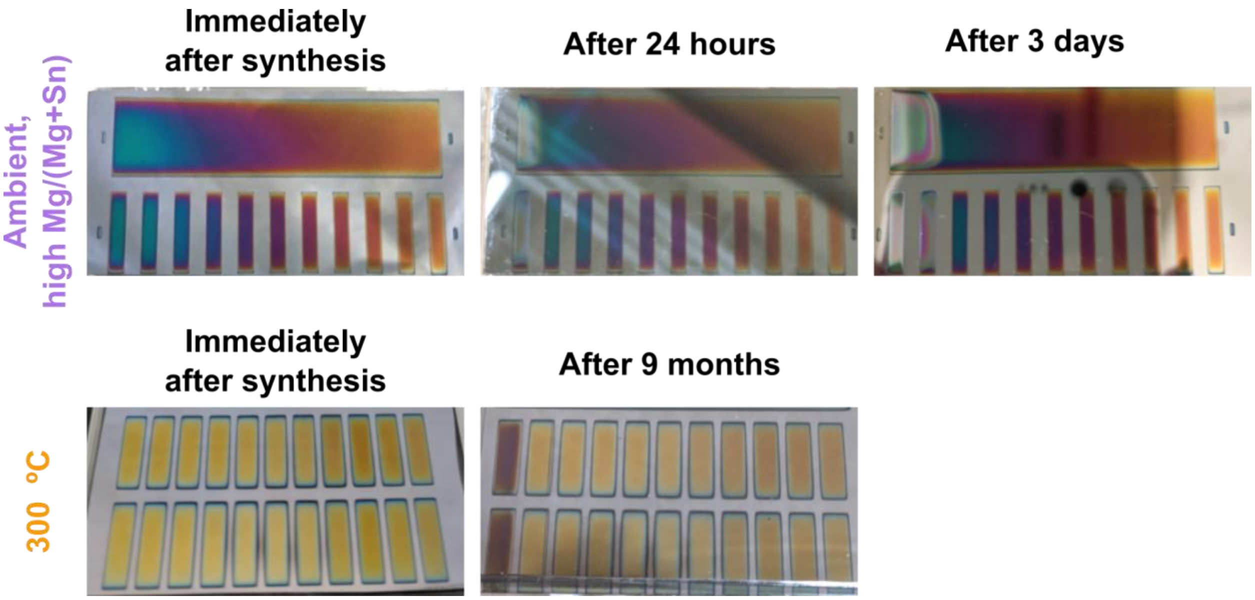

Figure S3: Photographs of sample libraries (with Mg-rich compositions on the left-hand side of each image) showing evolution of libraries with time. Top row: high Mg/(Mg+Sn) content library, which rapidly oxidized even in a N_2 environment. Bottom row: 300 ºC library showing a much smaller degree of oxidation after nine months. Library rows are 2 inches across.

Computational Investigation

Table S1: Predicted structures with space group name and number; polymorph energies compared across multiple levels of computation; optoelectronic structure with band gaps, absorption thresholds (α = 10^3 cm^{-1}) with indirect/forbidden transitions marked with *, electron and hole effective masses, and electronic and ionic dielectric contributions.

Polymorph Type	Space Group		Relative Energies (meV/at)			Optoelectronic Structure					
	Name	Number	PBE	SCAN	RPA	E_g (eV)	E at 10^3	m^*_e/m_0	m^*_h/m_0	$\varepsilon_e/\varepsilon_0$	$\varepsilon_i/\varepsilon_0$
Wurtzite	$Pna2_1$	33	0.0	0.0	0.0	2.47	2.60	0.22	2.4	4.8	4.3
	$Pmc2_1$	26	5.2	5.3	5.3	2.34	2.45	0.21	3.0	4.8	4.3
Zinc blende	$I\bar{4}2d$	122	18.1	21.1	25.4	2.33	2.42	0.21	3.7	4.9	4.2
	$P\bar{4}m2$	115	26.8	29.2	29.8	2.13	2.22	0.20	4.2	5.0	4.3
Rocksalt	$P2/c$	13	90.3	67.5	70.0	3.17	3.56*	0.22	5.5	5.5	17.2
	$I4_1/amd$	141	114.5	92.7	109.2	2.93	3.24*	0.23	7.7	6.0	21.5

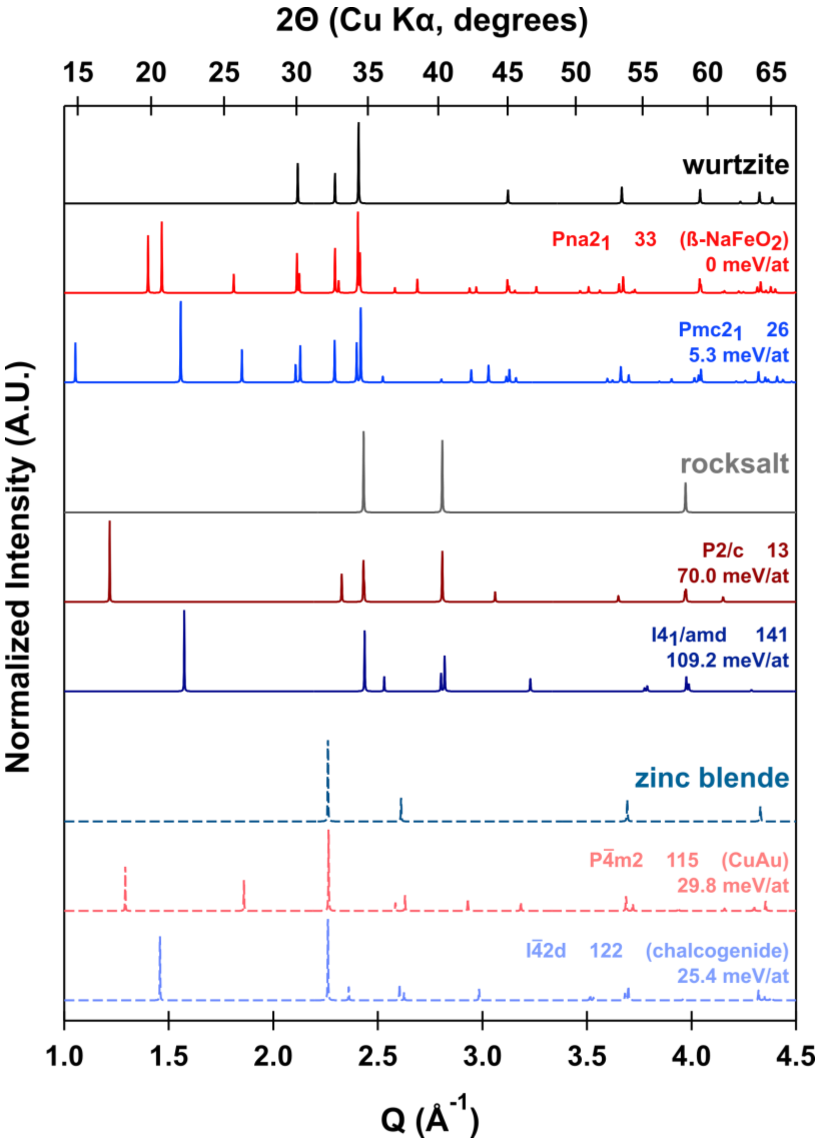

Figure S4: Calculated diffraction patterns for the three cation-disordered structure types for $MgSnN_2$, each compared with the two lowest-energy ordered structures of that type. Both Q and 2θ (Cu Kα) are given.

Optoelectronic Properties

Table S2: Hall effect measurement results for measurements performed at 40K.

Temperature (ºC)	Approximate Mg/(Mg+Sn)	Resistivity (Ω•m)	Carrier concentration (cm^{-3})	Mobility (cm^2/V•s)	Hall voltage (V)
**300**	0.5	4.65 × 10^{-2}	-7.5 × 10^{19}	1.79	-1.655 × 10^{-3}
**400**	0.4	9.91 × 10^{-2}	-3.4 × 10^{20}	0.19	-6.297 × 10^{-5}

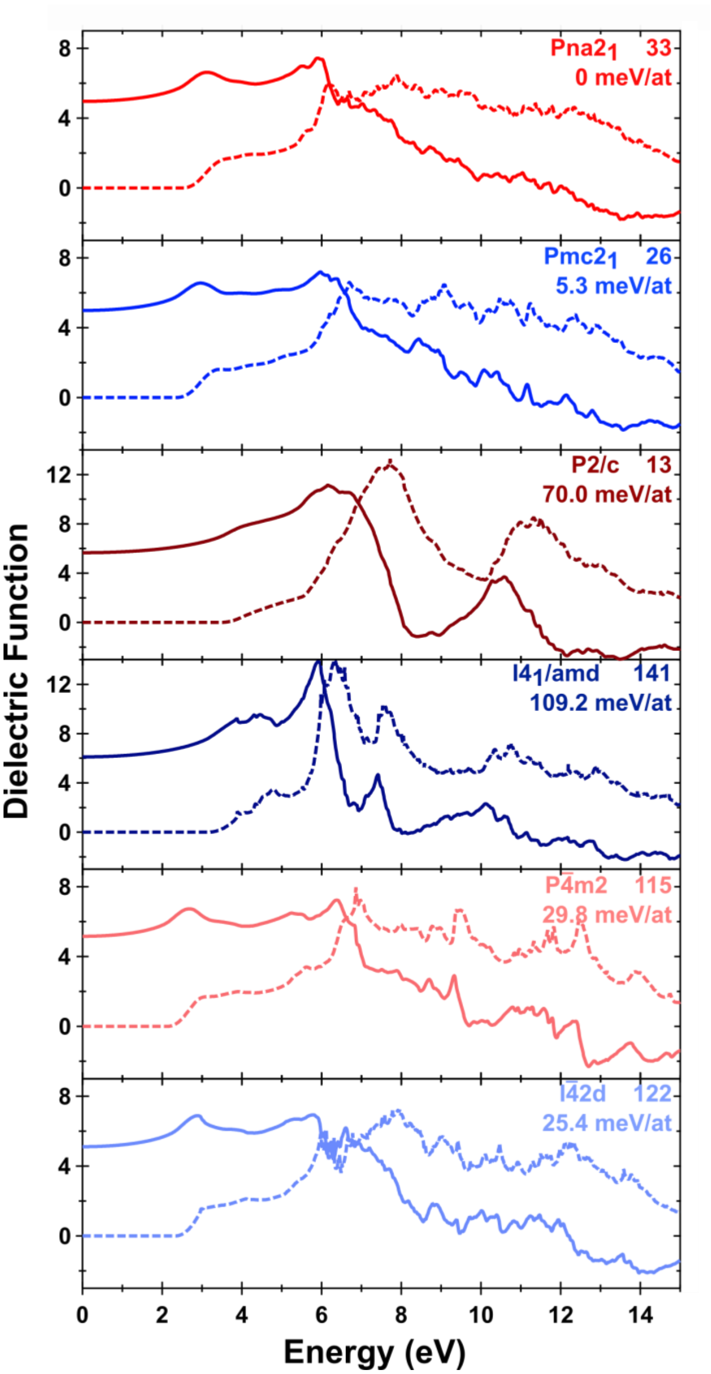

Figure S5: Calculated dielectric functions for the six lowest-energy ordered $MgSnN_2$ structures discussed in Figure 2, with ε_1 (solid traces) and ε_2 (dashed traces) indicated separately.

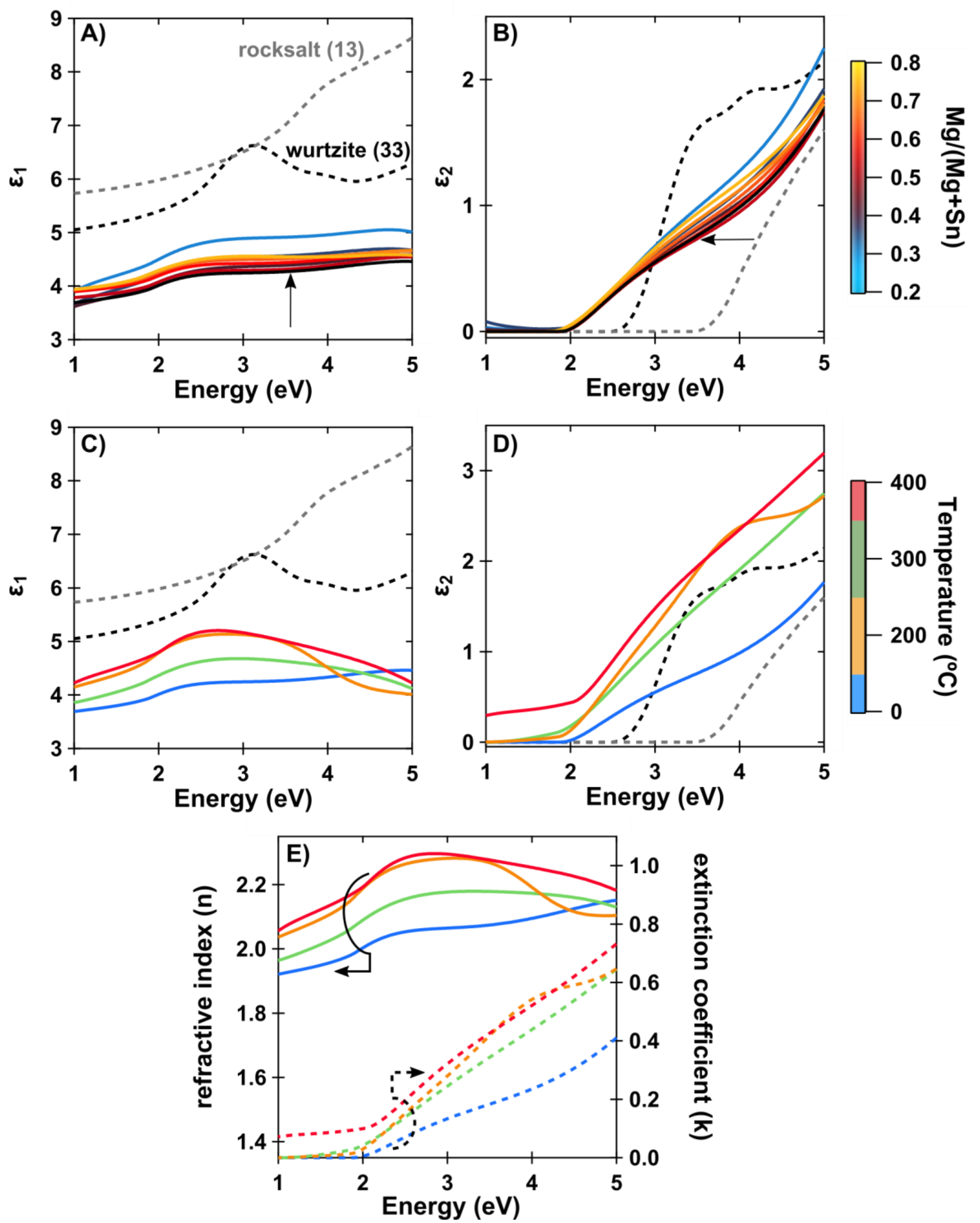

Figure S6: Additional optical properties calculated from spectroscopic ellipsometry. A) ε_1 and B) ε_2 for ambient temperature sample library presented in Figure 3A. The black arrows indicate the stoichiometric sample for this library. C) ε_1 and D) ε_2 for the Mg/(Mg+Sn) ≈ 0.5 samples presented in Figure 3B. Calculated ε_1 and ε_2 for the lowest-energy cation-ordered wurtzite (SG 33) and rocksalt (SG 13) are indicated with dashed lines in A-D. E) *n* (solid traces) and *k* (dashed traces) functions for the Mg/(Mg+Ss) ≈ 0.5 samples, using the same color scale as C) and D).

Crystallographic Information Files

Other supporting information includes CIFs for disordered wurtzite, rocksalt, and zinc blende-derived forms of $MgSnN_2$, as well as $MgSnN_2$ in SG 33, 26, 13, 141, 166, 122, and 115.
